# Editorial: Severe Eosinophilic Disorders: Mechanisms and Clinical Management

**DOI:** 10.3389/fimmu.2019.02118

**Published:** 2019-09-04

**Authors:** Praveen Akuthota, Josiane S. Neves, Shigeharu Ueki

**Affiliations:** ^1^Division of Pulmonary, Critical Care, and Sleep Medicine, University of California, San Diego, La Jolla, CA, United States; ^2^Federal University of Rio de Janeiro, Institute of Biomedical Sciences, Rio de Janeiro, Brazil; ^3^Department of General Internal Medicine and Clinical Laboratory Medicine, Akita University Graduate School of Medicine, Akita, Japan

**Keywords:** asthma, eosinophils, hypereosinophilia, IL-5, degranulation, rhinosinusitis

Severe eosinophilic disorders present a significant clinical challenge. This is due to several factors, including the varied etiologies, a high tendency to recur, and a frequent need to treat with long-term systemic steroids, accompanied by a host of short- and long-term side effects. Eosinophilic disorders can in certain circumstances be life threatening. Lessons from the recent emergence of anti-IL-5 monoclonal antibody therapies have highlighted the clinical benefits of eosinophil depletion. To promote improved understanding of the eosinophil, a pleiotropic multifunctional leukocyte, and associated eosinophilic diseases, potentially leading to advances in clinical management, we sought with this Research Topic both original research and review articles that were truly bench to bedside in their consideration of severe eosinophilic disease, encompassing studies of eosinophil cell biology and immunology, murine models of eosinophilic inflammation, and translational/clinical investigations.

## Eosinophil Extracellular Traps

Mukherjee et al. review a growing body of literature that delineates the ability of eosinophils to release DNA extracellular traps. These are similar to neutrophil extracellular traps, in that they may have important functions in defense against extracellular pathogens. An important consideration in this article is contextualizing the process of “ETosis” alongside the other canonical pathways for eosinophil degranulation.

Conversely, Ueki et al. (co-editor for this Research Topic) extend the discussion of the potential pathogenic role of eosinophil extracellular traps in the specific case of allergic bronchopulmonary aspergillosis (ABPA). In this entity, the accumulation of eosinophils and formation of eosinophil extracellular traps in response to fungal elements may thicken pathologic mucous and further perpetuate airway inflammation, representing a novel perspective into this eosinophilic lung disease.

## Piecemeal Degranulation

One of the other now canonical pathways for eosinophil degranulation is known as piecemeal degranulation (PMD). Dias et al. demonstrate in a murine model of *Schistosoma mansoni* infection that the eosinophils recovered from the liver were predominantly undergoing PMD. Their data also supported the release of the granule protein MBP by PMD, suggestive of well-coordinated, selective release of granule proteins by eosinophils during parasitic infection.

## *Ex vivo* Studies of Murine and Human Eosinophils

Amorim et al. reported on novel effects of leptin stimulation of human and murine eosinophils, extending knowledge on the emerging role of leptin in immune regulation. In their study, leptin was shown to activate leukotriene C_4_ synthesis with an increase in lipid body/lipid droplet formation through the sequential release of CCL5 and prostaglandin D_2_.

Comprehensive metabolic profiling by extracellular flux analysis from Porter et al. provides valuable insights into the metabolic flexibility of human eosinophils. The study indicates that while eosinophils have similar capacity for glycolysis in comparison to neutrophils, they have a greater ability to utilize glucose for oxidative metabolism. These findings have important implications in contextualizing the diverse immunoregulatory capacities of eosinophils.

## Murine Models of Allergic Airways Inflammation

Gubernatorova et al. report the results of conditional gene targeting of IL-6 in a house dust mite model of allergic asthma. IL-6 has been noted in human disease to be an important biomarker of severity by the Severe Asthma Research program [Peters et al. (1)]. The authors of the study in this Research Topic found that dendritic cells and macrophages were the sources of IL-6 and that ablation of IL-6 reduced eosinophilic inflammation, as did specific macrophage IL-6 ablation.

Two manuscripts in the Research Topic involved the role of non-apoptotic Fas signaling in resolution of allergic airways inflammation. In the first, Williams et al. show that non-apoptotic Fas signaling is key to the resolution of eosinophilic inflammation and that disruption of non-apoptotic Fas signaling results in prolonged eosinophilic airways inflammation. In the second manuscript, Ferreira et al. utilize T cell-specific Fas conditional knockout mice to show that antigen exposure during homeostatic T cell proliferation after non-lethal radiation in these animals was sufficient to induce prolonged eosinophilic airways inflammation. Together, these manuscripts highlight the likely importance of Fas signaling in resolution of Th2 inflammation.

## Clinical Considerations in Asthma and Rhinosinusitis

In the era of anti-IL5 therapies for asthma, it has been well-established that eosinophil-targeting strategies reduce asthma exacerbations. In their mini-review, Nakagome and Nagata discuss the potential roles of eosinophils in promoting asthma exacerbation, with a section of particular interest that details the ability of viral infection to augment eosinophil-mediated pathology.

Kobayashi et al. report a blinded, placebo controlled study testing a novel delivery of inhaled steroid to the upper airway in patients with chronic rhinosinusitis in the setting of asthma whose upper airway disease was refractory to the usual delivery method of intranasal steroid via the inhalation through the nares. In this study, inhaling beclomethasone through for delivery to the lower airways followed by exhalation through the nose for deposition to the upper airways led to radiographic and symptomatic improvements in the upper airway, suggesting that this alternative delivery strategy may be a viable therapeutic option.

Eotaxin-3 (CCL26) has been noted as an important epithelial-derived chemokine associated with an array of eosinophilic disease. Yamada et al. extend this growing body of work to establish circulating eotaxin-3 as a biomarker that correlates with eosinophil infiltration in tissue samples from chronic rhinosinusitis independent of blood eosinophil counts.

## Hypereosinophilia and Hypereosinophilic Syndromes

The evaluation of hypereosinophilia is challenging in pediatric patients and important distinctions compared to the evaluation of hypereosinophilia in adults exist. Schwartz and Fulkerson present their differential diagnosis and approach to the child presenting with hypereosinophilia.

There are common cytogenic abnormalities associated with myelodysplastic syndromes (MDS), but there are less well-characterized abnormalities that have been described. Rai et al. report on two cases of MDS with hypereosinophilia that led to fatal end organ damage, with both cases associated with the poorly characterized cytogenetic abnormality der(1;7)(q10;p10).

## Conclusions

Eosinophilic disorders represent a wide range of conditions that share the commonality of the eosinophil itself, allowing for studies of eosinophil biology to inform multiple disease entities and, in turn, for studies of one eosinophilic disorder to inform understand of others. The editors of this Research Topic believe that continuing to explore eosinophil biology and commonalities between eosinophilic diseases will continue be a fruitful endeavor.

## Author Contributions

PA drafted the manuscript. JN and SU have made a substantial, direct and intellectual contribution to the work. All authors provided approval for publication of the content.

### Conflict of Interest Statement

SU received honoraria and/or research grants from Maruho and AstraZeneca. PA has received consulting fees, honoraria, and research grants from GlaxoSmithKline, AstraZeneca, Projects in Knowledge, Prime Education, Advance Medical, and AKH. The remaining author declares that the research was conducted in the absence of any commercial or financial relationships that could be construed as a potential conflict of interest.
